# Prognostic Significance of SUVmax Combined With Lactate Dehydrogenase in Advanced Lung Cancer Patients Treated With Immune Checkpoint Inhibitor Plus Chemotherapy: A Retrospective Study

**DOI:** 10.3389/fonc.2021.652312

**Published:** 2021-05-18

**Authors:** Linping Ke, Lu Wang, Jinming Yu, Xue Meng

**Affiliations:** ^1^ Department of Clinical Medicine, Weifang Medical University, Weifang, China; ^2^ Department of Radiation Oncology, Shandong Cancer Hospital and Institute, Shandong First Medical University and Shandong Academy of Medical Sciences, Jinan, China; ^3^ Department of Radiation Oncology, School of Medicine, Shandong University, Jinan, China

**Keywords:** prognosis, hematology, immunotherapy, lung cancer, SUVmax

## Abstract

**Purpose:**

This research aims to investigate the predictive capacity of PET/CT quantitative parameters combined with haematological parameters in advanced lung cancer patients treated with immune checkpoint inhibitor (ICI) plus chemotherapy.

**Methods:**

A total of 120 patients who underwent ^18^F-fluorodeoxyglucose positron emission tomography/computed tomography (^18^F-FDG PET/CT) were enrolled before therapy. The following parameters were calculated: the maximum, mean, and peak standardized uptake value (SUVmax, SUVmean, and SUVpeak, respectively); total tumour volume (MTV) and total lesion glycolysis (TLG); and whole-body metabolic values (MTVwb, TLGwb, SUVmeanwb, and SUVmaxwb). Lactate dehydrogenase (LDH) levels, absolute neutrophil count, absolute platelet count, albumin levels and derived neutrophil to lymphocyte ratio (dNLR) were also computed. The associations between the variables and therapy outcome (evaluated by iRECIST) were analyzed.

**Results:**

Based on iRECIST, 32 of 120 patients showed iPD, 43 iSD, 36 iPR and 9 iCR. Multivariate analysis found that SUVmax, MTVwb, LDH and absolute platelet count were associated with treatment response (P =0.015, P =0.005, P <0.001 and P =0.015, respectively). Kaplan-Meier survival analyses showed that SUVmax ≥11.42 and LDH ≥245 U/L were associated with shorter OS (P = 0.001 and P = 0.004, respectively). Multivariate Cox regression revealed that SUVmax and LDH alone were not correlated with survival prognosis (p>0.05), but the combination of SUVmax and LDH was independently associated with OS (P=0.015, P=0.001, respectively). The median survival time (MST) for the low (LDH<245 and SUVmax<11.42), intermediate(LDH<245 or SUVmax<11.42), and high(SUVmax≥11.42 and LDH≥245) groups was 24.10 months (95% CI: 19.43 to 28.77), 17.41 months (95% CI: 15.83 to 18.99), and 13.76 months (95% CI: 12.51 to 15.02), respectively.

**Conclusion:**

This study identified that SUVmax plus LDH correlated with the survival outcome in patients with advanced lung cancer receiving PD-1/PD-L1 blockade plus chemotherapy.

## Introduction

Recently, GLOBOCAN reported that lung cancer has the highest rate of incidence and mortality for men and women in the world (1). It has a poor prognosis, with a 5-year survival rate of 15% (2, 3). Unfortunately, a large population of primary lung cancer patients are diagnosed at stage IV (4). The 5‐year survival rate of metastatic lung cancer is no more than 5% because of the lack of appropriate treatment options. Therefore, systemic therapy has become the primary treatment option.

Today, systemic therapy for advanced lung cancer mainly includes immunotherapy and chemotherapy, as well as their combination. Many preclinical studies have shown the immunomodulatory effects of cytotoxic chemotherapy. Whether for non-small-cell lung cancer (NSCLC) or small cell lung cancer (SCLC), chemotherapy combined with PD-1 receptor or ligand inhibitor plays an important role in first-line therapy, and this approach has been undertaken to improve treatment responses and prolong survival (5–11).

Immune checkpoint inhibitors (ICIs) plus chemotherapy are recommended as the optimal first‐line therapy for patients with advanced NSCLC (9). A meta-analysis (12) found that overall survival (OS) and progression-free survival (PFS) advantages of ICI therapies were observed in patients with NSCLC with low or high programmed cell death 1 ligand 1 (PD‐L1) expression levels but not in intermediate PD‐L1 TPS patients. Update data for the KEYNOTE-189 study found that regardless of PD-L1 positivity, both median OS and PFS improved in the pembrolizumab combination chemotherapy group in patients with metastatic NSCLC (13). Interestingly, the HRs (hazard ratios) for PFS were similar among PD-L1-expressing and PD-L1-negative patients (13). Therefore, PD‐L1 alone is not recommended as a molecular biomarker to identify eligible patients for immunotherapy plus chemotherapy in routine clinical practice.

According to research, the inflammation process is associated with the mechanism of oncogene signaling pathway activation and immunoresistance in the cancer population (14). Of note, a pro-inflammatory status is connected with poor outcomes in cancer patients (15–17). The hematological parameters circulating white blood cells, absolute neutrophil count, absolute platelet count, lactate dehydrogenase (LDH) level and derived neutrophil-to-lymphocyte ratio (dNLR; absolute neutrophil count/[white blood cell concentration − absolute neutrophil count]) have been proposed as potential inflammatory biomarkers in cancer patients and are also correlated with poor outcomes in several solid tumors (17–21).

As an advanced imaging examination, [^18^F]F-FDG PET/CT (18F-fluorodeoxyglucose positron emission tomography/computed tomography) is widely used for response monitoring and prognostication for locally advanced NSCLC (22–24). The study found that SUVpeak, MTV, and TLG has predictive significance in the response to immunotherapy in patients with melanoma (25).Another study showed that baseline MTVwb and SUVmean correlate with survival in advanced non-small cell lung cancer patients treated with pembrolizumab (26). At the same time, the entire tumor burden evaluated by 18F-FDG PET/CT was proved to be the Predictors to immunotherapy in patients with metastatic lung cancer (27). Soussan et al. found that SUVmax, SUVpeak, SUVmean, TLG were prognostic factors for EFS(event-free survival) in lung cancer after chemotherapy, but MTV is not (28).A study that included 60 patients with lung cancer who received chemotherapy alone, and finally found that the whole-body PET/CT parameters (MTV,TLG) significantly associated with overall survival. However, SUVmaxwb and SUVmeanwb were not statistically significant association with OS (29). The uptake of FDG by malignant tissues as well as in inflammatory disorders is quantified by various parameters of [^18^F]F-FDG PET/CT, such as the standardized uptake value (SUV) (30–32).

Published retrospective studies reported that a pro-inflammatory status was associated with poor outcomes of immunotherapy in melanoma patients (33, 34). Mezquita et al. found that combining a dNLR greater than 3 with LDH greater than the upper limit of normal (ULN) could identify advanced NSCLC patients who would have poor outcomes from immunotherapy (21). We hypothesized that combining baseline parameters of hematology and [^18^F] F-FDG PET/CT would be correlated with a poor outcome of ICI therapy combined with chemotherapy in patients with advanced lung cancer.

## Materials and methods

### Population

This retrospective study enrolled 120 patients with advanced lung cancer at our institute from January 2017 to January 2020 who underwent pretreatment ^18^F-FDG PET/CT before receiving combination treatment of ICI plus chemotherapy. The inclusion criteria were as follows: (1) histologically or cytologically proven lung cancer; (2) TNM stage IV in the American Joint Committee on Cancer (AJCC) 8^th^ (35) staging system; (3) Eastern Cooperative Oncology Group Performance Status of 0 to 1; (4) more than 4 cycles (3 weeks to a cycle) of ICI plus chemotherapy; and (5) more than 18 years old. Exclusion criteria:

This study was approved by the Institutional Review Board of the Shandong Cancer Hospital and Institute (Jinan, China). All patients provided informed consent before treatment.

### PET-CT Imaging

In the Department of Nuclear Medicine and PET-CT Centre, all patients had to have serum glucose levels less than 11 mol/L and at least 6 h of fasting before intravenous administration of 370 MBq (10 mCi) of FDG. After resting in a lounge chair for a minimum of 60 min, all patients underwent 5 min whole-body emission scanning from the skull base to the upper femur. PET images were obtained with a dedicated PET/CT scanner (GEMINI TF Big Bore; Philips Healthcare). Under 4.25 mm/slice axial sampling thickness and 0.8 s rotation speed per rotation, spiral CT was performed.

All subjects were asked to maintain tidal breathing during PET scanning. The images were reconstructed by ordered-subset expectation maximization (OSEM) after attenuation correction. Then, the corresponding PET and CT images, as well as fused PET/CT images, were observed on a dedicated workstation (Xeleris; GE Healthcare) in the transverse, coronal, and sagittal planes. [^18^F]F-FDG PET/CT scans for all patients were performed before they received the combined treatment.

### Image Analysis

Two experienced nuclear medicine physicians outlined the regions of interest (ROIs) separately according to 3-dimensional CT scans and PET/CT fusion images by using MIM software (MIM, 6.2.8, Cleveland, OH, USA). The automated contouring program was set to a fixed standardized uptake value (SUV) threshold of 2.5 (34–36). Under the fixed threshold, the maximum, mean, and peak standardized uptake values (SUVmax, SUVmean, and SUVpeak, respectively), as well as total tumour volume (MTV) and total lesion glycolysis (TLG), were acquired. The whole-body burden values of SUVmax, SUVmean, SUVpeak, MTV, and TLG were named SUVmaxwb, SUVmeanwb, MTVwb, and TLGwb, which were defined as their respective summations.

### Hematological Parameters

We also collected hematological parameters within 3 days before the start of combination treatment by searching the patient’s electronic medical records: lactate dehydrogenase (LDH) levels (the normal reference range was 109–245 U/L), absolute neutrophil count, absolute platelet count, albumin levels and dNLR [absolute neutrophil count/(white blood cell concentration − absolute neutrophil count)].

### Response Evaluation

Every subject’s best treatment response was evaluated by iRECIST (Immune Response Evaluation Criteria in Solid Tumors) (37) according to their every-6-weekly clinical and radiological follow-up. Patients were grouped as experiencing progression of disease (iPD), stable disease (iSD) or partial response or complete response (iPR/iCR). Clinical benefit (CB) was grouped as iPR or iCR, and no clinical benefit (no-CB) was grouped as iPD or iSD.

### Statistical Analyses

Overall survival (OS) was computed as the start of combination therapy until death for any reason or the date of the last follow-up. To summarize the results of this study, descriptive statistics are reported as mean ± standard deviation. Statistical analysis tried to solve several objectives. The independent sample Student’s t-test was used for continuous variables. Kaplan-Meier analyses and the log-rank test were used to quantify the associations with survival. Cox proportional hazards regression aimed to distinguish variables independently correlated with survival. Statistically significant variables in the univariate analysis (P <0.10) were included in the final multivariate model. Logistic regression analysis with the upward elimination method was performed to further verify the relationships found. Cut-off points were obtained by receiver operating characteristic (ROC) curve analysis. SPSS Statistics version 26.0 (IBM Corporation, Armonk, NY, USA) was used for statistical analyses. P value < 0.05 was considered statistically significant.

## Results

### Characteristics of Patients

**Table 1** shows the patient demographics and baseline characteristics.

**Table 1:** Baseline characteristics of all 120 patients.

Characteristic	Total (n=120)	CB (Clinical Benefit) (n=45)	no-CB (no-Clinical Benefit) (n=75)
Age (years)			
Median age, year (range)	60 (37-81)	60 (43-76)	60 (37-81)
Gender n. (%)			
Male	83 (69.2)	35 (77.8)	48 (64.0)
Female	37 (30.8)	10 (22.2)	27 (36.0)
Performance status (ECOG) n. (%)			
0,1	120 (100)	45 (100)	75 (100)
≥2	0 (0)	0 (0)	0 (0)
Histology n. (%)			
Small cell lung cancer	33 (27.5)	11 (24.4)	22 (29.3)
Non-Small cell lung cancer			
Squamous cell carcinoma	33 (27.5)	13 (28.9)	20 (26.7)
Non-Squamous cell carcinoma	54 (45.0)	21 (46.7)	33 (44.0)
Previously treated n. (%)	34 (28.3)	14 (31.1)	20 (26.7)
Previous therapy n. (%)			
Thoracic radiotherapy	26 (21.7)	11 (24.4)	15 (20.0)
Target therapy	15 (12.5)	7 (15.6)	8 (10.7)
Chemotherapy	32 (26.7)	12 (26.7)	20 (26.7)
Smoking status n. (%)			
Former or current	60 (50.0)	29 (64.4)	31 (41.3)
Never	60 (50.0)	16 (35.6)	44 (58.7)
PD-L1 expression n. (%)			
≥1%	34 (28.3)	14 (31.1)	20 (26.7)
<1%	39 (32.5)	12 (26.7)	27 (36.0)
NA	47 (39.2)	19 (42.2)	28 (37.3)

ECOG, Eastern Cooperative Oncology Group; PD-L1, programmed death ligand 1, was acquired by immunohistochemistry; NA, not available.

This retrospective study comprised 120 patients. The patients were predominantly male (69.2%, 83/120), with a median age of 60 years (range 37–81 years) at the time of diagnosis. Thirty-five (77.8%) of 45 patients were male in the CB group, and 48 (64.0%) of 75 patients were male in the no-CB group. Out of 120 patients, 33 had small cell lung cancer (SCLC), 33 had squamous cell lung carcinoma, and 54 had non-squamous-cell lung carcinoma. They had predominantly NSCLC, at 21 (46.7%) and 33 (44%) in the CB group and no-CB group, respectively. The ECOG performance status for all patients was 0 or 1. Before starting ICI plus chemotherapy, 26 patients received thoracic radiotherapy, 15 received targeted therapy, and 32 patients received chemotherapy. Some 28.3% (34/120) of the sample showed a PD‐L1 tumour proportion score (TPS) ≥1% by immunohistochemical analysis, and 32.5% (39/120) showed a PD‐L1 TPS <1%. The smoking status was similar in the two groups: 29 (64.4%) patients in the CB group were former or current smokers, versus 31 (41.3%) in the no-CB group. Staging was carried out on the basis of the 8th edition of the American Joint Committee on Cancer tumour, node and metastasis staging system.

### Radiological Outcome and Prognosis

Radiological follow-up was available in all patients. Based on iRECIST, 32 of 120 patients showed iPD, 43 iSD, 36 iPR and 9 iCR. The proportion of patients in the CB group was 37.5% (45/120), and that in the no-CB group was 62.5% (75/120). The difference in outcomes that were continuous variables between the CB group and no-CB group, as calculated by the independent-sample Student’s t-test, is shown in **Table 2**.

**Table 2:** Independent sample student t-test of groups difference (CB vs no-CB).

Parameters	no-CB (n=75) (mean ± SD)	CB (n=45) (mean ± SD)	P value
SUVmax	12.83 ± 5.31	10.82 ± 5.27	0.046^*^
SUVpeak	9.43 ± 5.15	7.75 ± 4.12	0.052
SUVmean	5.24 ± 2.16	5.25 ± 1.95	0.982
MTV	92.56 ± 131.68	46.22 ± 61.22	0.010^*^
TLG	713.35 ± 822.01	342.10 ± 734.80	0.055
SUVmaxwb	22.13 ± 13.72	18.80 ± 21.58	0.302
SUVmeanwb	6.55 ± 2.93	6.82 ± 2.32	0.600
MTVwb	97.53 ± 120.14	53.38 ± 59.93	0.009^*^
TLGwb	495.88 ± 668.78	271.28 ± 576.55	0.066
LDH,U/L	291.96 ± 106.70	204.84 ± 59.85	<0.001^*^
Absolute neutrophil count,×10^9^/L	5.03 ± 3.04	4.40 ± 1.24	0.113
Absolute platelet count,×10^9^/L	239.56 ± 70.24	201.44 ± 78.10	0.007^*^
Albumin levels, g/L	43.71 ± 3.97	43.85 ± 3.78	0.851
dNLR	2.43 ± 1.30	2.67 ± 1.46	0.360

no-CB, no-Clinical Benefit, was defined as complete or partial response; CB, Clinical Benefit, was defined as stable disease or progressive disease response; SUVmax, maximum standardized uptake value; SUVpeak, peak standardized uptake value; SUVmean, mean standardized uptake value; MTV, metabolic tumor volume; TLG, total lesion glycolysis; SUVmaxwb, whole-body maximum standardized uptake value; SUVmeanwb, whole-body mean standardized uptake value; MTVwb, whole-body metabolic tumor volume; TLGwb, whole-body total lesion glycolysis; LDH, lactate dehydrogenase; dNLR was defined as absolute neutrophil count/[white blood cell concentration − absolute neutrophil count];* p<0.05.

Patients in the CB group had a significantly lower SUVmax, MTV, MTVwb, LDH and absolute platelet count than patients in the no-CB group (10.82 ± 5.27 vs 12.83 ± 5.31, p=0.046; 46.22 ± 61.22 vs 92.56 ± 131.68, p=0.010; 53.38 ± 59.93 vs. 97.53 ± 120.14, p=0.009; 204.84 ± 59.85 vs. 291.96 ± 106.70, p<0.001; 201.44 ± 78.10 vs. 239.56 ± 70.24, p=0.007, respectively) (**Table 2**). In multivariate analysis, SUVmax, MTVwb, LDH and absolute platelet count were associated with progressive disease, with an OR of 4.44 (95% CI, 1.33-14.80; P =0.015), 12.63 (95% CI, 2.17-73.56; P =0.005), 22.20 (95% CI, 6.31-78.05; P <0.001), and 4.85(95% CI, 1.60-14.76; P =0.015), respectively (**Table 3**).

**Table 3:** Logistic regression of clinical benefit in 120 patients.

Variables	OR (95% CI)	P value
Gender(vs)		
Male		
Female	0.67 (0.12-3.68)	0.643
Age		
<60		
≥60	1.15 (0.38-3.48)	0.805
Histology		
Squamous NSCLC		
Non-squamous NSCLC		
Small cell lung cancer	1.26 (0.60-2.62)	0.545
Smoking status		
Never		
Former or current	0.15 (0.03-0.77)	0.024^*^
PD-L1 expression		
<1%		
≥1%		
NA	0.15 (0.03-0.77)	0.701
SUVmax		
<11.42		
≥11.42	4.44 (1.33-14.80)	0.015^*^
MTV		
<40.36		
≥40.36	0.99 (0.22-4.41)	0.990
MTVwb		
<61.45		
≥61.45	12.63 (2.17-73.56)	0.005^*^
LDH,U/L		
<245		
≥245	22.20 (6.31-78.05)	<0.001^*^
Absolute platelet count,×10^9^/L		
<177		
≥177	4.85 (1.60-14.76)	0.005^*^

OR, odds ratio; NSCLC, non‒small-cell lung cancer; PD-L1, programmed death ligand 1, was acquired by immunohistochemistry; NA, not available; SUVmax, maximum standardized uptake value; MTV, metabolic tumor volume; MTVwb, whole-body metabolic tumor volume; LDH lactate dehydrogenase; * p<0.05.

### Survival Analysis

At the time of analysis, 76 patients (63.3%) had died. The median follow-up time was 15.40 months (range, 1.37–29.43 months). The estimated median survival time (MST) for the entire cohort was 16.67 months (95% CI: 15.41–17.93 months), with estimated 6-month, 12-month and 18-month OS rates of 96.6%, 80.0% and 41.9%, respectively.

Univariate analysis of OS among patients with advanced lung cancer revealed that smoking status, SUVmax and LDH were significant predictors, whereas PD-L1 expression, sex, age, histology, SUVmaxwb, SUVpeak, SUVmean, SUVmeanwb, MTV, MTVwb, TLG, TLGwb, absolute platelet count, absolute neutrophil count, albumin levels and dNLR were not significant prognostic factors (**Table 4**). Based on the ROC curve, the optimal cut-off value of SUVmax was 11.42.

**Table 4:** Univariate analysis of overall survival in all patients.

Variables	OS	
	HR (95% CI)	P value
Gender (Male vs Female)	0.730 (0.387-1.379)	0.332
Age (<60 vs ≥60)	0.624 (0.355-1.096)	0.101
Histology (Squamous NSCLC vs Non-squamous NSCLC vs Small cell lung cancer)	1.213 (0.576-2.554)	0.612
Smoking status (Never vs Former or current)	1.385 (0.387-1.379)	0.025^*^
PD-L1 expression (<1% vs ≥1% vs NA)	1.206 (0.620-2.343)	0.581
SUVmax	1.059 (1.005-1.117)	0.033^*^
SUVmaxwb	1.010 (0.998-1.022)	0.119
SUVpeak	1.030 (0.975-1.089)	0.293
SUVmean	1.077 (0.920-1.261)	0.357
SUVmeanwb	0.998 (0.881-1.131)	0.981
MTV	1.000 (0.997-1.002)	0.864
MTVwb	0.999 (0.881-1.131)	0.486
TLG	1.000 (0.998-1.003)	0.987
TLGwb	1.000 (0.999-1.000)	0.906
LDH,U/L	1.003 (1.000-1.005)	0.049^*^
Absolute platelet count,×10^9^/L	0.999 (0.996-1.003)	0.660
Absolute neutrophil count,×10^9^/L	0.968 (0.850-1.103)	0.628
Albumin levels, g/L	0.968 (0.900-1.040)	0.374
dNLR	0.974 (0.791-1.199)	0.803

HR, hazard ratio; NSCLC non‒small-cell lung cancer; PD-L1, programmed death ligand 1, was acquired by immunohistochemistry; NA, not available; SUVmax maximum standardized uptake value; SUVpeak peak standardized uptake value; SUVmean mean standardized uptake value; MTV metabolic tumor volume; TLG total lesion glycolysis; SUVmaxwb whole-body maximum standardized uptake value; SUVmeanwb whole-body mean standardized uptake value; MTVwb, whole-body metabolic tumor volume; TLGwb, whole-body total lesion glycolysis; LDH lactate dehydrogenase; dNLR was defined as absolute neutrophil count/[white blood cell concentration − absolute neutrophil count]; OS, overall survival;* p<0.05.

**Figure 1:**
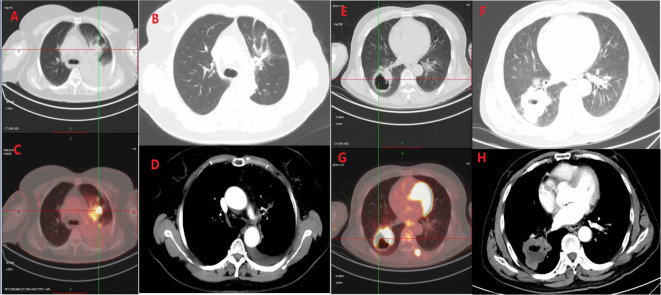
**(A)** A 63-year-old female patient with stage IVa left upper lobe adenocarcinoma. PET-CT before immunization and chemotherapy showed a SUVmax of 11.0 and an LDH of 196 **(A, C)**. The imaging efficacy evaluation after 6 cycles of treatment showed PR **(B, D)**. **(B)** A 58-year-old male patient with squamous cell carcinoma of the right lower lobe, PET-CT showed SUVmax of 22.8, LDH of 196 **(E, G)**, and disease progression **(F, H)** after 4 cycles of treatment.

According to the above, SUVmax and LDH were combined for analysis and were used to classify the patients into 3 groups: the high group (SUVmax≥11.42 and LDH≥245), 2 risk factors; intermediate group (LDH<245 or SUVmax<11.42), 1 risk factor; and low group (LDH<245 and SUVmax<11.42), 0 risk factors (**Figure 1**).

**Figure 2:**
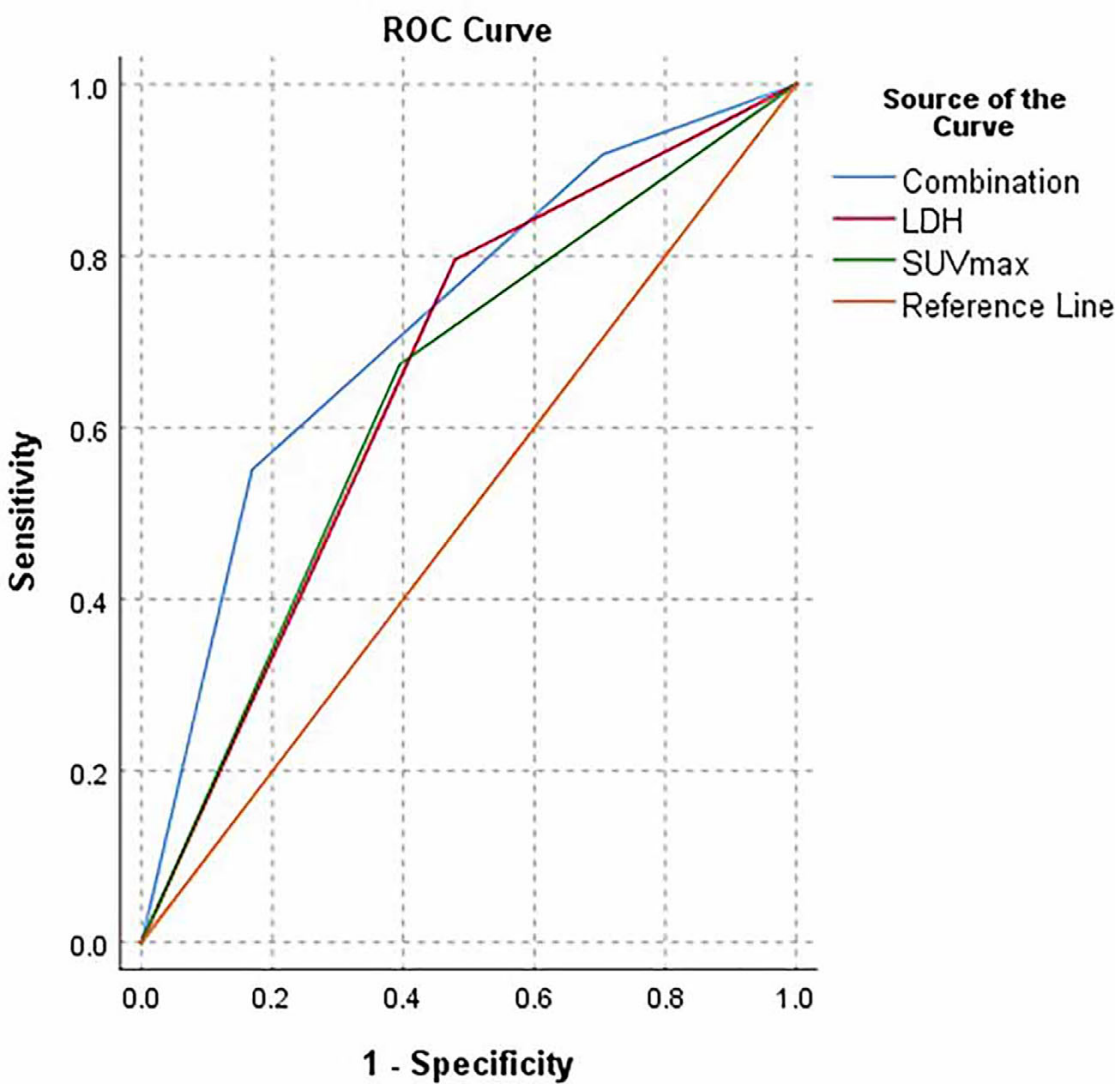
ROC curve of LDH (<245 VS ≥245), SUVmax(<11.42 vs ≥11.42), Combination group (Low group, LDH<245 and SUVmax<11.42; intermediate group, LDH<245 or SUVmax<11.42; high group, SUVmax≥11.42 and LDH≥245), respectively.

We further analyzed these selected parameters and found that the AUC of the combination of SUVmax and LDH was greater than that of SUVmax or LDH alone, with values of 0.723, 0.640 and 0. 659, respectively (**Figure 2**).

**Figure 3:**
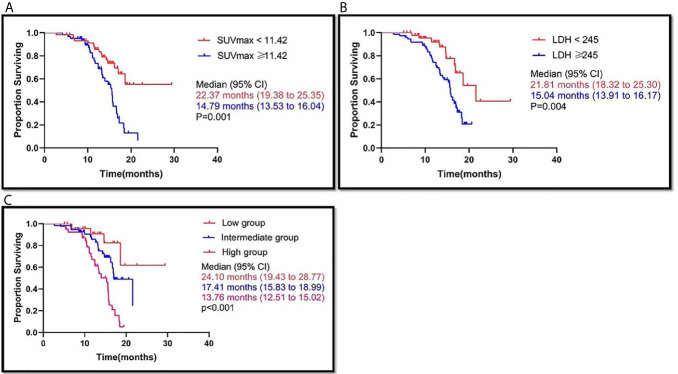
SUVmax≥11.42, LDH≥245 and high group (SUVmax≥11.42 and LDH≥245), was associated with poor outcome. Kaplan-Meier analysis for Overall survival (OS) in the SUVmax ≥11.42 vs. SUVmax <11.42 **(A)**; LDH ≥245 vs. LDH <245 **(B)**; high group (SUVmax≥11.42 and LDH≥245) vs. intermediate group (LDH<245 or SUVmax<11.42) vs. low group (LDH<245 and SUVmax<11.42) **(C)**, respectively.

The difference in MST between SUVmax ≥11.42 patients and SUVmax <11.42 patients was statistically significant (P=0.001), and the median (95% CI) survival time was 14.79 (13.53 to 16.04) months in the SUVmax ≥11.42 group and 22.37 (19.38 to 25.35) months in the SUVmax <11.42 group (**Figure 3A**). LDH ≥245 and LDH <245 were also associated with different MSTs (P=0.004). The median (95% CI) survival time was 15.04 (13.91 to 16.17) months in the LDH ≥245 group and 21.81 (18.32 to 25.30) months in the LDH <245 group (**Figure 3B**).

Out of all the patients, there were 25 patients in the low group, who had a median survival time of 24.10 months (95% CI: 19.43 to 28.77). There were 56 patients in the intermediate group, and their MST was 17.41 months (95% CI: 15.83 to 18.99). The 39 patients in the high group had an MST of 13.76 months (95% CI: 12.51 to 15.02). The difference in MST between these groups in the study was statistically significant (p<0.001) (**Figure 3C**).

In multivariate analysis, an independent prognostic factor associated with OS was the combination of primary tumour SUVmax and LDH (the high group had HR 2.397, 95% CI 0.808-7.112, P=0.015; the intermediate group had HR 6.399; 95% CI 2.201-18.602; P =0.001) (**Table 5**).

**Table 5:** Multivariate analysis of overall survival in all patients.

Characteristics	OS	
	HR (95%CI)	P value
Combination groups		
Low group		
Intermediate group	2.397 (0.808-7.112)	0.015^*^
High group	6.399 (2.201-18.602)	0.001^*^

OS, overall survival; HR, hazard ratio; * p<0.05.

### Subgroup Analysis

**Figure 4:**
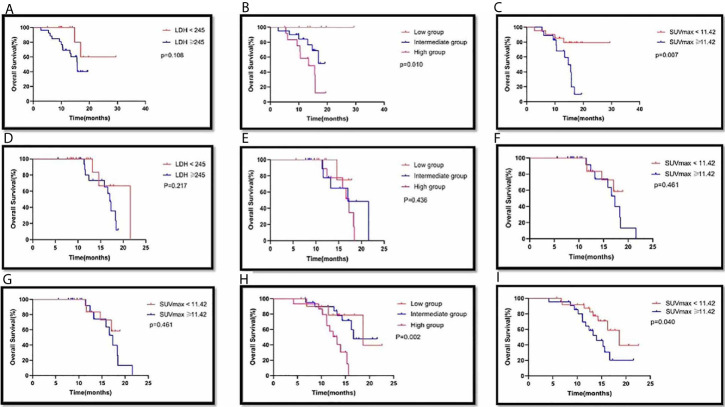
Survival curves of LDH(<245 VS ≥245), combination group(Low group, LDH<245 and SUVmax<11.42; intermediate group, LDH<245 or SUVmax<11.42; high group, SUVmax≥11.42 and LDH≥245) and SUVmax(<11.42 vs ≥11.42) of primary tumor in PD-L1 expression <1% **(A,B,C)**, PD-L1 expression ≥1% **(D,E,F)** and PD-L1 expression not available **(G,H,I)** with advanced lung cancer, respectively.

Among the 39 patients with PD-L1 expression <1%, there were 13 patients in the LDH <245 group, with an MST of 23.98 months (95% CI: 18.09-29.86), and 26 patients in the LDH ≥245 group, with an MST of 14.50 months (95% CI: 12.44-16.55). There was no significant difference in MST between the two groups (p=0.108) (**Figure 4A**). There were 7 patients in the low group, and none died; of the 20 patients in the intermediate group, 6 died; of the 12 patients in the high group, 9 died. There was a significant difference in MST among the different groups (p=0.010). There were 21 patients in the SUVmax <11.42 group, with an MST of 25.05 months (95% CI: 21.18-28.92); there were 18 patients in the SUVmax ≥11.42 group, with an MST of 13.62 months (95% CI: 11.65-15.59). There was a significant difference in MST between the two groups (p=0.007) (**Figure 4C**).

In Cox multivariate analysis, the only independent prognostic factor associated with OS was SUVmax (HR 4.359, 95% CI: 1.373-13.837, p=0.012)

Among the 34 patients with PD-L1 expression ≥1%, there were 16 patients in the LDH <245 group, with an MST of 19.02 months (95% CI: 15.47-22.58). The 18 patients in the LDH ≥245 group had an MST of 16.06 months (95% CI: 14.64-17.48). There was no significant difference in MST between the two groups (p=0.217). There were 7 patients in the low group, with an MST of 16.66 months (95% CI: 14.74-18.57); 15 patients in the intermediate group, with an MST of 17.51 months (95% CI: 14.20-20.83); and 12 patients in the high group, with an MST of 16.21 months (95% CI: 14.47-17.95). There was no significant difference in MST among the different groups (p=0.436). There were 13 patients in the SUVmax <11.42 group, with an MST of 16.95 months (95% CI: 15.31-18.59), and 21 patients in the SUVmax ≥11.42 group, with an MST of 16.66 months (95% CI: 14.74-18.57). There was no significant difference in MST between the two groups (p=0.461). (**Figure 4F**)

Among the 47 patients whose PD-L1 expression was not available, there were 18 patients in the LDH <245 group, with an MST of 18.20 months (95% CI: 15.25-21.16), and 29 patients in LDH ≥245 group, with an MST of 14.61 months (95% CI: 12.83-16.38). There was no significant difference of MST between the two groups (p=0.064). There were 11 patients in the low group, with an MST of 18.21 months (95% CI: 14.51-21.92); 21 patients in the intermediate group, with an MST of 17.51 months (95% CI: 17.45); and 15 patients in the high group, with an MST of 12.43 months (95% CI: 10.81-14.05). There was a significant difference in MST among the different groups (p=0.002). There were 25 patients in the SUVmax <11.42 group, with an MST of 17.75 months (95% CI: 15.25-20.25), and 22 patients in the SUVmax ≥11.42 group, with an MST of 14.31 months (95% CI: 12.12-16.51). There was a significant difference in MST between the two groups (p=0.040). In Cox multivariate analysis, independent prognostic factors associated with OS included combination group. Taking the low group as a reference, the HR of the high group was 5.356 (95% CI: 1.336-21.466, p=0.018), and the HR of the intermediate group was 1.144 (95% CI: 0.292-4.481, p=0.005) (**Figure 4I**).

**Figure 5:**
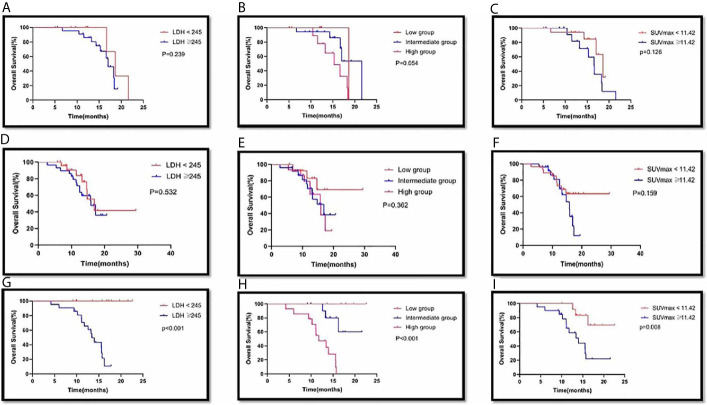
Survival curves of LDH(<245 VS ≥245), combination group(Low group, LDH<245 and SUVmax<11.42; intermediate group, LDH<245 or SUVmax<11.42; high group, SUVmax≥11.42 and LDH≥245) and SUVmax(<11.42 vs ≥11.42) of primary tumor in Squamous NSCLC **(A,B,C)**, Non-Squamous NSCLC **(D,E,F)** and SCLC **(G,H,I)** with advanced lung cancer, respectively.

In 33 patients with squamous non–small-cell lung cancer, there were 10 patients in the LDH <245 groups, with an MST of 18.96 months (95% CI: 16.17-21.75). There were 23 patients in the LDH ≥245 group, and their MST was 16.19 months (95% CI: 14.74-17.64). The difference in MST between these two groups was not statistically significant (p=0.239) (**Figure 5A**). There were 5 patients in the low group, with an MST of 18.63 months (95% CI: 18.63-18.63). There were 18 patients in the intermediate group, and their MST was 18.61 months (95% CI: 16.01-21.20). There were 10 patients in the high group, and their MST was 15.28 months (95% CI: 13.18-17.38). The difference in MST between these different groups was not statistically significant (p=0.054). There were 18 patients in the SUVmax <11.42 group, with an MST of 17.35 months (95% CI: 15.73-18.98), and 15 patients in the SUVmax ≥11.42 group, with an MST of 16.10 months (95% CI: 13.96-18.25). There was no significant difference in MST between the two groups (p=0.126). (**Figure 5C**)

In 54 patients with non-squamous non–small-cell lung cancer, there were 25 patients in the LDH <245 group, with an MST of 20.16 months (95% CI: 15.29-25.03). There were 29 patients in the LDH ≥245 group, and their MST was 15.27 months (95% CI: 13.23-17.30). The difference in MST between these two groups was not statistically significant (p=0.532). There were 14 patients in the low group, with an MST of 23.99 months (95% CI: 18.72-29.25). There were 25 patients in the intermediate group, and their MST was 15.32 months (95% CI: 12.97-17.67). There were 15 patients in the high group, and their MST was 14.75 months (95% CI: 12.60-16.90). The difference in MST between these different groups was not statistically significant (0.362). There were 28 patients in the SUVmax <11.42 group, with an MST of 22.38 months (95% CI: 18.63-26.13), and 26 patients in the SUVmax ≥11.42 group, with an MST of 14.52 months (95% CI: 12.90-16.14). There was no significant difference in MST between the two groups (p=0.159). (**Figure 5F**)

In 33 patients with small cell lung cancer, there were 12 patients in the LDH <245 group, and no patients died. Twenty-one patients were included in the LDH ≥245 group. The difference in MST between these two groups was statistically significant (p<0.001). There were 6 patients in the low group, 13 patients in the intermediate group, and 14 patients in the high group. The difference in MST between these different groups was statistically significant (p<0.001). There were 13 patients in the SUVmax <11.42 group, with an MST of 20.10 months (95% CI: 17.70-22.51), and 20 patients in the SUVmax ≥11.42 group, with an MST of 14.07 months (95% CI: 11.67-16.48). There was a significant difference in MST between the two groups (p=0.008). In the Cox multivariate analysis, neither SUVmax nor LDH nor SUVmax plus LDH was an independent prognostic factor for OS. (all p>0.05).

## Discussion

The tumour metabolic activity assayed by the advanced noninvasive examination method ^18^F-FDG PET/CT plays an important role in the diagnosis, staging, and evaluation of the treatment response and prognosis of lung cancer. Although some studies have shown the value of ^18^F-FDG PET/CT in immunotherapy for advanced lung cancer (27, 38), few data are available about its potential utility in the response evaluation of immunotherapy plus chemotherapy. It is worth mentioning that our study explored the prognostic evaluation and curative effect of SUVmax and LDH in patients treated with ICI plus chemotherapy. SUVmax ≥11.42 and LDH ≥245 were associated with significantly shorter OS (HR, 2.397, 95% CI, 0.808-7.112, P=0.015 and HR, 6.399; 95% CI, 2.201-18.602; P =0.001, respectively). No OS differences were observed in our cohort according to histology (SCLC/squamous NSCLC/nonsquamous NSCLC).

Our findings show that among all enrolled ^18^F-FDG PET imaging parameters and hematological parameters at baseline, SUVmax and LDH predicted the response to PD-L1/PD-1 blockade plus chemotherapy. This study suggested that a higher SUVmax or LDH at baseline led to a worse response to therapy, which implies that they may be promising parameters for the identification of patients who have a higher chance of not responding to ICI plus chemotherapy. Our findings likely reflect the potential correlation between inflammatory biology and the steady-state biological activity of tumors in response to combination therapy of ICI plus chemotherapy. ^18^F-FDG PET/CT is usually used to monitoring the response to immunotherapy or chemotherapy (39–42). A preliminary analysis found that the SUVmax at baseline was significantly different between responders to immunotherapy among patients with NSCLC (43). However, a prospective study of 32 patients (44) suggested that pretreatment SUVmax as shown by ^18^F-FDG PET/CT was not able to predict the response to chemotherapy in advanced NSCLC patients. These findings indicate that the presence of areas of high metabolic activity in tumors, which may be associated with histological differentiation (45), predicts PD-1/PD-L1 inhibitor activity at baseline and consequently the response to PD-1 blockade, but not to chemotherapy.

In our study, univariate analysis showed that not only SUVmax but also LDH was an independent prognostic factor for OS, and patients with higher SUVmax (≥11.42) or LDH (≥245) had a significantly shorter median survival time (P=0.001, P=0.004, respectively) than patients with lower SUVmax (<11.42) or LDH (<245), respectively. Previous studies also confirmed the prognostic significance of the SUVmax on ^18^F-FDG PET/CT in patients with lung cancer (45–48). However, some studies have not found the same (49–51).

After adjusting for age, sex, smoking status, PD-L1 expression, and histology, both SUVmax and LDH were not independent prognostic factors. The role of SUVmax in lung cancer remains controversial. A previous study revealed that the SUVmax of the primary tumour before treatment had no prognostic value in patients with locally advanced NSCLC (52). In the present study, subgroup analysis found that among people with PD-L1 expression less than 1, SUVmax was an independent prognostic factor for OS.

SUVmax has following limitations. First, it gives a single-pixel value representing the most intense ^18^F-FDG uptake by the tumour and may not be a strong surrogate marker of tumour biology (53). It may not reveal the heterogeneous nature of the tumour, and it is affected by statistical noise and pixel size (54). Furthermore, SUVmax is affected by a variety of factors, such as the physique of the subject, the level of blood glucose, and the time after the injection when imaging is done. Therefore, further large-sample and multicentre studies are needed to confirm our conclusions.

Previous studies revealed that inflammatory status was associated with worse prognosis in advanced disease patients treated with chemotherapy (55–57). Several studies have demonstrated that elevated LDH was significantly associated with shorter survival (21, 58–60). Our study found that in patients with advanced lung cancer, the median (95% CI) survival times of patients with LDH ≥245 U/L and LDH <245 U/L were 15.04 months (95% CI: 13.91 to 16.17) and 21.81 months (95% CI: 18.32 to 25.30), respectively. These times were not significantly different. We suppose that combining SUVmax with LDH can provide a more accurate prediction of prognosis than SUVmax or LDH alone.

Our findings show that the combination of SUVmax and LDH was an independent prognostic factor for survival, with patients in the high group (SUVmax≥11.42 and LDH≥245) being more likely to have progressive disease (P =0.001) and having a shorter survival time (median, 13.76 months) than those in the intermediate or low group (P <0.05). The risk of death was 6.339 times and 2.397 times higher in the high and intermediate groups, respectively, than in the low group (LDH<245 and SUVmax<11.42). In our subgroup analysis, the combination of SUVmax and LDH was also an independent prognostic factor of survival for patients whose PD-L1 expression was not available. Based on the above results, we concluded that this combination was a predictor of poor outcome from ICI plus chemotherapy. SUVmax plus LDH may be more relevant to prognosis than SUVmax or LDH alone because it not only reflects tumour metabolic activity but also reflects the inflammatory status. SUVmax, which is a functional metabolism biomarker derived from ^18^F-FDG PET, can be used to estimate the survival value in a noninvasive manner. Additionally, SUVmax reflects metabolic activity in malignant cancerous cells, which has been significantly correlated with PD-1/PD-L1 status and CD8^+^ tumour-infiltrating lymphocytes (TILs) (61). One study showed that high pretreatment LDH levels were significantly associated with lower overall survival (62). Although the associations had statistical significance, there is no doubt that the relationship between SUVmax or LDH alone and the survival prognosis is relatively weak, reflecting that additional, uncertain factors exist, impossibly explained by the combination of these two markers.

Our study has several limitations. First, the sample size was relatively small. Second, this study was a retrospective analysis and hence cannot exclude potential biases. Third, ^18^F-FDG is not a cancer-specific imaging molecule, especially for outcome evaluation during immunotherapy. The question is whether ICIs are related to inflammatory responses, which stimulate neutrophils and macrophages and activate T cells around the tumour site. The high metabolism aroused by immune cells makes this radiotracer inadequately specific. Further research with a larger number of subjects is needed to verify our conclusions. Fourth, because of different histologies and various therapeutic methods, patients are highly heterogeneous, which limits the applicability of our results. Last but not least, both hematologic parameters and metabolic parameters are often influenced by other, uncontrollable factors; therefore, some underlying confusion cannot be avoided. Nonetheless, these weaknesses do not decrease the contributions of our research or minimize the significance of the factors associated with survival.

## Conclusion

This study identified both a new noninvasive imaging-based biomarker and a hematological parameter that were correlated with poor outcomes after PD-1/PD-L1 blockade plus chemotherapy in patients with advanced-stage NSCLC. The negative correlation between combined therapy and the FDG-avid tumour metabolism parameters expressed by SUVmax could reveal intratumoural necrotic and/or apoptotic changes, and LDH could show a relationship with tumour inflammation. Further investigation is needed to confirm these possible associations and to elucidate the role of SUVmax plus LDH as a predictor of clinical response to targeted anti-PD-1/PD-L1 therapy combined with chemotherapy in a larger number of patients.

## Data Availability Statement

The original contributions presented in the study are included in the article/supplementary material. Further inquiries can be directed to the corresponding authors.

## Ethics Statement

The studies involving human participants were reviewed and approved by Shandong Cancer Hospital affiliated to Shandong University Ethical Committee. The patients/participants provided their written informed consent to participate in this study.

## Author Contributions

XM and JY contributed conception and design of the study. LK organized the database. LK performed the statistical analysis. LK, XM, and JY wrote the first draft of the manuscript. All authors contributed to the article and approved the submitted version.

## Funding

This study is supported by the Innovation Project of Shandong Academy of Medical Sciences (2019-04), and the Academic Promotion Program of Shandong First Medical University (2019ZL002), National Natural Science Foundation of China (81972864), Academic Promotion Program of Shandong First Medical University (2019RC002), Science and Technology Support Plan for Youth Innovation Teams of Universities in Shandong Province (2019KJL001) and Science and Technology Plan of Jinan (201907113).

## Conflict of Interest

The authors declare that the research was conducted in the absence of any commercial or financial relationships that could be construed as a potential conflict of interest.
